# A new method for detecting unilateral spatial neglect with tracing tasks using the Rey-Osterrieth complex figure: a pilot study

**DOI:** 10.1007/s10072-024-07540-6

**Published:** 2024-05-08

**Authors:** Rintaro Ohama, Shuji Matsumoto, Yumi Ohama, Katsuya Yokoyama, Megumi Shimodozono

**Affiliations:** 1 Department of Rehabilitation and Physical Medicine, Kagoshima University Graduate School of Medical and Dental Sciences, 8-35-1, Sakuragaoka, Kagoshima City, Kagoshima, 890-8520 Japan; 2 Centre for Medical Sciences, Ibaraki Prefectural University of Health Sciences, Inashiki-Gun, Ibaraki, Japan

**Keywords:** Stroke, Visuospatial agnosia, Constructional apraxia, Working memory, Attention, USN

## Abstract

**Purpose:**

To explore efficacy of the “Rey-Osterrieth complex figure (ROCF) tracing task” as a new test to detect unilateral spatial neglect (USN).

**Methods:**

Subjects were 40 healthy control (HC) and 20 right brain-damaged patients with (USN + , n = 10) or without USN (USN − , n = 10). After the ROCF copying task, the tracing task was performed under conditions that did not leave any tracing lines on the sample figure. Evaluation used the conventional 36-point scoring system, laterality index (LI) as the ratio of the left and right structure scores, and the number of overlaps for each of the left and right structures scored.

**Results:**

In the tracing task, USN + showed a lower LI than HC. Furthermore, left-sided neglect was sometimes more evident than in the copying task. Regarding the total overlapping score, USN + showed a greater score than HC. The right-sided overlapping scores in USN + and USN − were also greater than that in HC. In the right brain-damaged subjects, clinically meaningful correlations were not found between evaluations in the ROCF tracing task and in conventional USN screening tests. Receiver-operating-characteristic analysis to test the power of detection showed moderate performance for the tracing LI (AUC = 0.76, 95% CI = 0.54–0.97), which was greater than that of other tests. Further, the total overlapping score in the tracing task showed sensitivity 0.9 (highest among the tests performed), specificity 0.5, and AUC 0.68 (95% CI = 0.43–0.92).

**Conclusion:**

The ROCF tracing task might be a convenient method to detect USN and to reveal the extent of spatial working memory impairment.

## Introduction

Unilateral spatial neglect (USN) is defined as an impairment in reporting, responding to, or localizing stimuli presented to the contralateral side of a brain lesion [1], often occurring as left USN due to right-side brain damage [2]. USN manifests clinically as unilateral spatial inattention and trunk tilt, and it indicates poor prognosis due to an increased risk of falls [3], reduced activities of daily living (ADL) [4], and worsened quality of life [5], as well as an increased need for care after discharge [6] compared to patients without USN.

USN encompasses various subtypes, including egocentric and allocentric neglect [7], body space to distal space [8], and those related to visual search and time-related responses [9], which are assumed to be related to the site of injury [10] and affected by different mechanisms of onset [11, 12]. In a systematic review of the incidence of USN after stroke, Esposito et al. [13] reported that USN is seen in 45% of patients with right-side brain damage in the acute phase and in 20% of those in the chronic phase, but the prevalence of USN varies among studies. It has been pointed out that USN occurrence after stroke may be underestimated [14]. It is therefore recommended that a combination of cancellation, bisection, and copying tests be assessed, corresponding respectively to egocentric, allocentric, and egocentric/allocentric neglect [10, 15]. On the other hand, deficits in spatial working memory from posterior parietal cortex damage have been hypothesized to be involved in existence and severity of USN through the observation of ‘productive manifestations’ or “revisiting behavior” [16, 17]. In clinical practice, a simple and reliable testing method is needed to determine USN subtypes and guide clinicians to provide effective treatment according to the subtype. However, few methods have been reported for detecting USN in light of deficits in spatial working memory [16, 17, for the review see 10]. Wojciulk et al. [18] reported a new cancellation task that detects a behavior of repeated cancellation of items aggravated by the absence of visible cancellation marks.

We previously noted that some patients with right-side brain damage exhibit more USN symptoms in the tracing task—tracing without leaving tracing lines directly on the sample figure— than in the traditional copying task, as well as characteristic symptoms of repeatedly tracing the same lines over and over again. According to the phenomenon observed in the tracing task, patients with USN might not be able to remember where they have already traced and where they have not yet traced. On the other hand, the Rey-Osterrieth complex figure test (ROCF) [19, 20] is a neuropsychological assessment that is widely used as a copying or memorization task for which quantitative evaluation methods have been established. Therefore, we conceived the idea that ROCF could be used to quantify the characteristic phenomena that occur in the tracing task.

In the present pilot study, we propose “ROCF tracing task” as a new convenient method to detect USN and explore the method’s potential efficacies by assessing its correlation with conventional USN screening tests and its diagnostic utility.

## Methods

This cross-sectional pilot study was designed to examine the characteristics of the tracing task and its potential use in clinical situations. To assess whether the “ROCF tracing task” can be used to identify USN, patients with right brain damage were divided into two groups according to presence or absence of USN based on screening tests. A group of healthy subjects was also included. To examine characteristics of the ROCF tracing task, the tracing task and a copy task [21] were performed using ROCF (i.e. the “ROCF copy task”) and scores were compared among the three groups. In addition, correlations between scores on the tracing task and scores on conventional USN screening methods (the cancellation test, line bisection test, and ROCF copy task) were computed. Finally, to characterize performance of the ROCF tracing task in terms of sensitivity and specificity, a receiver operating characteristic (ROC) curve was computed for each test in the two groups of patients with right brain damage.

### Subjects

Subjects with right brain damage were recruited from patients admitted to Kirishima Rehabilitation Center of Kagoshima University Hospital. Healthy subjects were recruited from the general public.

The number of patients was set at 20 on the basis of cross-sectional studies of patients with right brain damage [22, 23].

The sample size of the healthy subject group was set at 40, double the number of right brain-damaged subjects, on the basis of cross-sectional studies of healthy controls and patients with right-brain damage [24, 25]. Thus, the patient and control sample sizes were different.

### Inclusion and exclusion criteria

Inclusion criteria for patients with right brain damage were that they had had a first stroke, were right-handed, and were aged 20–80 years. Right brain damage was determined by consulting the medical history and by using medical information from the hospital where acute treatment was carried out, as well as by computed tomography images of the brain. Time since stroke onset was not considered in the patient inclusion criteria. Inclusion criterion for healthy subjects was being of age 20–80 years.

Exclusion criteria for both patients with right-brain damage and healthy subjects were a history of treatment for brain disorders (in the case of patients with right-brain-damage, a history of illness like stroke, brain injury, or surgery to the brain prior to the current stroke), mental illness or dementia which could affect the assessment results, or inability to understand the test description due to the effects of higher brain dysfunction or other factors.

We administered to participants various tasks described below, after obtaining written informed consent. This study complied with the Declaration of Helsinki and was approved by the Clinical Research Ethics Committee of Kagoshima University Hospital, Faculty of Medicine and Dentistry (No: 24–140).

### USN screening

Presence of USN was determined by impaired performance on the USN screening test. The USN screening test consists of the line cancellation test [26], star cancellation test [27], line bisection test [27], and copying a landscape constructed of five objects [15] this is the method of assessment used in our hospital [28]. Cutoff values for the line bisection test and star cancellation test were based on the Japanese version of the Behavioral Inattention Test. The line cancellation test was judged by two or more omissions on the left half of the sheet compared with the number of omissions on the right half. The copying test was judged by omission of at least one detail of the left part. Patients with right-brain damage who showed symptoms of USN in any of the tests were assigned to the “USN + group”; other patients with right-brain damage were assigned to the “USN − group”.

### Test and evaluations for the ROCF tracing and copying tasks

**Fig. 1:**
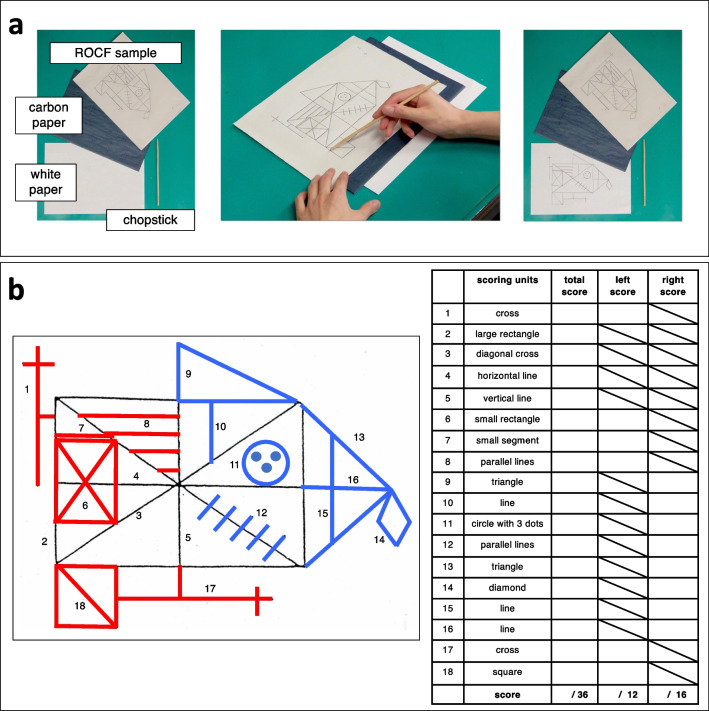
**a** ROCF tracing task; **b** Scoring respective 18 units of the ROCF and estimating the total, left (red), and right (blue) scores. Copying and tracing scores were estimated after the ROCF copying and tracing tasks were completed by the subjects

The ROCF tracing task was conducted immediately after the ROCF copying task. In the tracing task, subjects were presented with the ROCF sample and a sheet of carbon paper overlaid on a sheet of white paper, and instructed to trace the ROCF with chopsticks so as not to leave any tracing lines directly on the sample figure (Fig. 1a). At the same time, they were instructed not to trace the same spot more than once.

Both the tracing and copying tasks were evaluated conventionally with a maximum score of 36 points across 18 scoring units [29]. In addition, to identify differences between left and right structure copying and tracing, the left and right units were scored separately (Fig. 1b) (the left and right maximum scores were 12 and 16 points, respectively). In addition, the left–right ratio of correct responses was calculated as the Laterality Index (LI):

$$\mathrm{Laterality\;Index }\;({\text{LI}}) = \frac{(\mathrm{left\;construction\;score }/12)}{(\mathrm{right\;construction\;score }/16)}$$

For example, an LI below 1 indicates a greater proportion of correct responses in the right structures, and an LI above 1 indicates a greater proportion of correct responses in the left structures.

Further, to score the frequencies of overlapping (i.e., tracing the same item) in the respective copying and tracing tasks, each scoring unit of the ROCF structures was scored 1 point for a complete overlap and 0.5 points for an incomplete overlap, and was calculated as the total, left, and right “overlapping scores” by adding the number of times the unit was traced with an overlap.

### Procedure

Subjects were first interviewed about their education history and medical history related to the exclusion criteria, and tested using the Mini Mental State Examination-Japanese (MMSE-J) [30, 31]. The ROCF copying task and tracing task were then administered, on the same day.

The rules of the ROCF tracing task were explained to the subjects immediately after they had performed the ROCF copying task, and then they carried out the ROCF tracing task. If it was deemed that a subject did not understand the rules of the tracing task, the subject was asked to stop the task immediately and restart it after the rules were explained again. A subject was dismissed after having completed all of the tasks, and we then scored each task.

### Statistical analyses

We compared right and left scores, LIs, and overlapping scores on the ROCF copying and tracing tasks among the three groups: healthy, USN − , and USN + . Primary outcome was the LIs on the ROCF tracing task, and the secondary outcome was the total overlapping score on the ROCF tracing task. The others were exploratory outcomes. The Shapiro–Wilk test was used to assess whether variables approximately followed a normal distribution; if lack of normality was judged, non-parametric tests (Kruskal–Wallis ANOVA followed by the Mann–Whitney U test with the Holm correction) were employed. A *p*-value of < 0.05 was considered to be statistically significant for the primary outcome. The effect size (*r*) was also calculated as *r* = √(t^2^/(t^2^ + df)).

To characterize the ROCF tracing task compared with the conventional USN screening test for subjects with right brain damage, we evaluated the correlations between scores or LI in the ROCF tracing task and those in the star cancellation test, line bisection test, or ROCF copying task, using Spearman's rank correlation coefficient. Furthermore, we performed Receiver Operating Characteristic (ROC) curve analysis to explore the performance, via the area under the ROC curve (AUC), of the scores and LI both in the ROCF tracing task as a USN detection test and in the conventional USN screening tests (i.e. the star cancellation test, line bisection test and the ROCF copying task). Tests with AUC greater than 0.9 were interpreted as highly accurate, 0.7–0.9 as moderately accurate, and 0.5–0.7 as rather inaccurate; an AUC of 0.5 represents a chance result [32].

All statistical analyses were performed using R (The R Foundation for Statistical Computing, version 4. 0. 2).

## Results

The examinations were carried out with 40 healthy volunteers and 20 patients with right-brain damage: 10 patients were in the USN + group and the other 10 were in the USN − group. The characteristics of each group are presented in Table 1. There were no extreme differences in age, gender, school attendance, or MMSE-J scores among the three groups, nor were there any between the USN + and USN − groups in duration from onset or proportion of cerebral infarction and hemorrhage diagnoses.

**Table 1:** Demographic characteristics of the subjects

		Healthy group (n = 40)	USN– group (n = 10)	USN + group (n = 10)	*p* value
Age (years)		61 (48.5–70.2)	56 (46.4–59.6)	55.7 (9.9)	0.17^1)^
Gender (F/M)		19/21	2/8	2/8	0.26^2)^
School attendance (years)		12 (12–16)	13 (12–16)	14.5 (12–16)	0.63^1)^
Mini Mental State Examination		30 (30–30)	29 (28–29.5)	29 (27.5–30)	0.71^1)^
Duration from onset (weeks)		–	32 (5.75–271)	26 (13.2–156)	0.92^3)^
Diagnosis (infarction/ hemorrhage)		–	2/8	3/7	1^2)^
lesion sites	frontal lobe	–	1	2	–
	parietal lobe	–	0	2	–
	temporal lobe	–	1	4	–
	radiate crown	–	2	1	–
	basal ganglia	–	5	7	–
	thalamus	–	4	2	–

USN–, absence of unilateral spatial neglect (USN) based on USN screening tests after right brain injury; USN + , presence of USN based on USN screening tests after right brain injury. Data for each task are presented as the median (first quartile–third quartile). For brain-damage lesions, in cases with multiple sites of damage, all relevant damaged lesions were included in the counts

^1)^Kruskal–Wallis ANOVA, ^2)^Fisher’s exact test, ^3)^Mann–Whitney U test

**Fig. 2:**
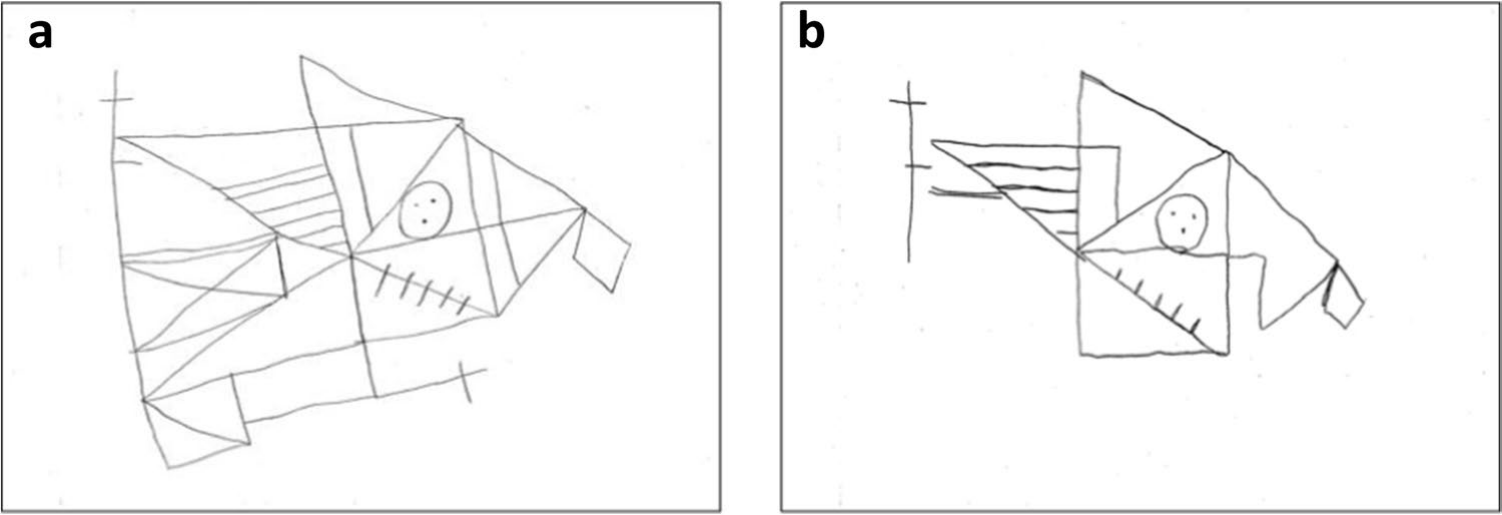
Example of the ROCF “copying” (**a**) and “tracing” tasks (**b**) by a patient in the USN + group

Figure 2 shows an example of the performance by a patient with USN in the ROCF copying task (Fig. 2a) and the tracing task (Fig. 2b). He obtained a total of 32 points and an LI of 0.83 in the copying task, and a total of 24 points and an LI of 0.72 in the tracing task. In case of overlapping scores, while he obtained 0 points in total for the copying task, the right score was 1.5 points, the left score was 1 point, and the total score was 2.5 points for the tracing task.

### ROCF copying task scores and LI

The ROCF copying scores and LI in the healthy, USN − , and USN + groups are shown in Table 2. Both the USN + group and the USN − group had lower copying scores than the healthy controls, but little difference were found between the USN + and USN − groups. On the other hand, USN + had an LI that was smaller than that in the healthy controls; little differences in LI were found between the USN + and USN − groups.

**Table 2:** Rey-Osterrieth complex figure test (ROCF) copying-task scores and Laterality Index (LI) in the three groups: Healthy, USN–, USN +

	Healthy control	USN–	USN +	^a^ANOVA *p* value	^*b*^*Post-hoc* *p* value	Effect size *r*
Copying Score	36.0 (35.0–36.0)	34.0 (32.5–35.5)	33.5 (32.2–35.5)	0.01	0.04^1)^	0.32^1)^
					0.03^2)^	0.35^2)^
					0.81^3)^	0.04^3)^
Copying LI	1.0 (0.99–1.0)	0.91 (0.83–0.92)	0.84 (0.83–0.97)	0.01	0.53^1)^	0.15^1)^
					< 0.001^2)^	0.42^2)^
					0.50^3)^	0.14^3)^

Data for each task are presented as the median (first quartile–third quartile)

USN–, absence of unilateral spatial neglect (USN) group; USN + , presence of USN group

^a^Kruskal-Wallis ANOVA, ^b^Post-hoc test using the Holm correction, ^1)^Healthy control vs. USN–; ^2)^Healthy control vs. USN + ; ^3)^USN– vs. USN +

### ROCF tracing task scores and LI

Table 3 shows the summary of the ROCF tracing scores and LI in the 3 groups. While there were no significant differences among the 3 groups in the tracing scores, a significant between-group difference was seen in LI: while the USN − group did not differ significantly from the healthy controls, the USN + group showed a smaller LI than the controls (*p* < 0.001, *r* = 0.42). The median LI for the USN + group was 0.84 (interquartile range [IQR] 0.75–0.90) and for the USN– group was 0.86 (IQR 0.86–0.97), and subjects in the USN + group lost more points on the left side of the ROCF tracing task than subjects in the USN– group. Clinically, in a few patients with or without USN, left-sided neglect was more pronounced in the tracing than in the copying task.

**Table 3:** ROCF tracing-task scores and Laterality Index (LI) in the three groups: Healthy, USN–, USN +

	Healthy control	USN–	USN +	^a^ANOVA *p* value	^*b*^*Post-hoc* *p* value	Effect size *r*
Tracing Score	34.0 (32.0–36.0)	33.0 (30.0–34.0)	33.0 (31.0–34.0)	0.11	0.25^1)^	0.21^1)^
					0.27^2)^	0.23^2)^
					0.93^3)^	0.02^3)^
Tracing LI	1.0 (0.91–1.0)	0.86 (0.86–0.97)	0.84 (0.75–0.90)	0.009	0.66^1)^	0.05^1)^
					< 0.001^2)^	0.42^2)^
					0.09^3)^	0.48^3)^

Data for each task are presented as the median (first quartile–third quartile)

USN–, absence of unilateral spatial neglect (USN) group; USN + , presence of USN group

^a^Kruskal-Wallis ANOVA, ^b^Post-hoc test using the Holm correction, ^1)^Healthy control vs. USN–; ^2)^Healthy control vs. USN + ; ^3)^USN– vs. USN +

### Overlapping scores for the ROCF copying task and tracing task

The summary of the overlapping scores for the copying task and tracing task in the 3 groups are shown in Table 4. In the ROCF copying task, there were no significant differences in the right, left, or total overlapping scores among the 3 groups. In the ROCF tracing task, however, the right, left, and total overlapping scores evidenced possible significant differences among the 3 groups. In post-hoc pairwise comparisons, the right overlapping score was greater in the USN + and USN − groups than in the controls. On the contrary, the left overlapping score in the USN + group was greater than in the controls, but not that in the USN − group. The left overlapping score in the USN − group was slightly greater than in the controls. For the total overlapping score, only the USN + group showed a significantly greater score than in the controls.

**Table 4:** Overlapping scores (right, left and total) for the three groups in the ROCF copying task and tracing task

		Healthy control	USN–	USN +	^a^ANOVA *p* value	^*b*^*Post-hoc* *p* value	Effect size *r*
Copying Task	Right	0.0 (0.0–0.0)	0.0 (0.0–0.0)	0.0 (0.0–0.0)	0.68	–	–
	Left	0.0 (0.0–0.0)	0.0 (0.0–0.0)	0.0 (0.0–0.0)	0.71	–	–
	Total	0.0 (0.0–0.0)	0.0 (0.0–0.37)	0.0 (0.0–0.0)	0.59	–	–
Tracing Task	Right	0.0 (0.0–0.0)	1.0 (0.25–1.37)	2.0 (1.12–2.37)	< 0.001	< 0.001^1)^	0.46^1)^
						< 0.001^2)^	0.64^2)^
						0.13^3)^	0.32^3)^
	Left	0.5 (0.0–1.0)	1.5 (0.62–1.50)	1.75 (1.00–2.50)	0.001	0.06^1)^	0.29^1)^
						< 0.001^2)^	0.47^2)^
						0.33^3)^	0.30^3)^
	Total	1.5 (0.50–2.12)	2.0 (1.12–3.37)	3.25 (2.50–4.87)	0.006	0.20^1)^	0.18^1)^
						0.003^2)^	0.43^2)^
						0.33^3)^	0.32^3)^

Data for each task are presented as the median (first quartile–third quartile)

USN–, absence of unilateral spatial neglect (USN) group; USN + , presence of USN group

^a^Kruskal-Wallis ANOVA, ^b^Post-hoc test using the Holm correction, ^1)^Healthy control vs. USN–; ^2)^Healthy control vs. USN + ; ^3)^USN– vs. USN +

### Correlation and diagnostic utility of the ROCF tracing task with conventional USN screening tests

Correlations between the ROCF tracing task and the conventional USN screening tests are shown in Table 5. Clinically meaningful correlations were not found between the ROCF tracing task and conventional USN screening tests.

**Table 5:** Spearman’s rank correlations between evaluations in the ROCF tracing task and those in conventional USN screening tests such as the star cancellation test, line bisection test, and the ROCF copying task among patients with right-brain damage (n = 20)

	ROCF tracing task				
	Score	LI	Right overlapping score	Left overlapping score	Total overlapping score
Star cancellation	0.09	0.25	0.006	–0.22	–0.11
Line bisection	0.07	0.11	–0.01	–0.11	–0.11
ROCF copying score	0.54	0.50	–0.15	–0.23	–0.18
ROCF copying LI	0.21	0.29	–0.19	–0.27	–0.26

LI, laterality index

Due to the small sample size, we judged correlation is not clinically meaningful if it is not greater than 0.8

A summary of performance analysis based on ROC curves for evaluations in the ROCF tracing task and conventional USN detection task is shown in Table 6. Specificity and sensitivity of each task for a chosen threshold (cutoff point) are shown along with values of AUC for ROC curves with varying cutoff points. Cutoff points for each task were chosen by the point on the ROC curve closest to the upper left-hand corner (100% sensitivity and 100% specificity) [32]. Confidence intervals for AUC were computed by DeLong’s method [33]. In the conventional USN detection tasks (such as the cancellation test, line bisection test, and the copying score), sensitivities were 0.5 or less, although they showed relatively high specificity of 0.7 or more. In contrast, in the ROCF tracing task, tracing LI showed sensitivity 0.8, specificity 0.7; AUC was 0.76, implying that is moderately accurate (95% CI = 0.54–0.97), and had the highest AUC (best performance) among the tests performed. Further, total overlapping score showed 70% beyond the cutoff point, the sensitivity 0.9, specificity 0.5; AUC 0.68 (95% CI = 0.43–0.92), and had the highest sensitivity among the tests performed.

**Table 6:** Receiver Operating Characteristic (ROC) curve analysis for evaluations (independent variables) in the ROCF tracing task and those in the conventional USN detection task among patients with right-brain damage (n = 20)

	Cutoff point	%beyond cutoff point	Sensitivity	Specificity	AUC (95% CI)
Star cancellation test	< 53.9	20	0.40	1.00	0.70 (0.54–0.86)
Line bisection test	< 8.51	25	0.40	0.90	0.66 (0.47–0.85)
ROCF copying score	< 33.8	40	0.50	0.70	0.53 (0.26–0.79)
ROCF copying LI	< 0.95	60	0.70	0.50	0.58 (0.33–0.83)
ROCF tracing score	< 30.5	30	0.20	0.60	0.49 (0.22–0.75)
ROCF tracing LI	< 0.93	55	0.80	0.70	0.76 (0.54–0.97)
Right overlapping score	> 1.25	50	0.70	0.70	0.69 (0.44–0.94)
Left overlapping score	> 1.98	35	0.50	0.80	0.62 (0.36–0.88)
Total overlapping score	> 1.75	70	0.90	0.50	0.68 (0.43–0.92)

AUC, area under the ROC curve; CI, confidence interval; LI, laterality index

## Discussion

In this study, we compared the performance of the copying task and the tracing task using the ROCF in three groups: a healthy control group, a USN − group (absence of USN based on USN screening after right-side brain damage), and a USN + group (presence of USN after right-side brain damage).

ROCF copying scores among the healthy group, the USN − group, and the USN + group differed in clinical meaningful ways; however, differences in ROCF tracing scores among the three groups were not clinically meaningful. In addition, both the USN + and USN − groups (i.e., patients with right-side brain damage) showed lower copying scores than did the healthy controls, but not lower tracing scores. Four main cognitive processes have been postulated to be active in figure copying: visual and spatial analysis, drawing plan preparation, execution, and control processes [34]; previous studies comparing brain activity in copying and tracing in healthy subjects have reported stronger activation in the bilateral interparietal sulci, premotor cortex, and supplementary motor cortex in copying [35]. Constructional apraxia often occurs in patients with right-side brain damage and impairs their ability to discern precise spatial relationships between the components of an object [36], making it difficult to copy drawings. It has been reported that tracing is possible even in the presence of constructional apraxia [37], so the ROCF tracing task was expected to be easier than the copying task. Thus, the USN + and USN − groups could have ROCF tracing scores comparable to those of healthy controls, but not so for copying scores. In total, however, we should consider several factors that influence the difficulties of the copying and tracing scores: (1) the copying task was carried out immediately before the tracing task; (2) the participants were instructed not to trace the same lines repeatedly; and (3) the task condition of the present tracing, which did not leave tracing lines after tracing, increased the burden on the motor working memory.

In the ROCF tracing task, the LI in the USN + group was significantly smaller than that in the control group, and was somewhat smaller than that in the USN − group. Leyland et al. [38] reported that when patients with USN were presented with two portraits in a row and asked to copy and trace them, they showed allocentric neglect symptoms in copying and egocentric neglect symptoms in tracing. The present results with the ROCF tracing task suggest that the nature of the ROCF, in which each unit is scored according to its shape and position, reduced the number of errors due to constructional apraxia after right-side brain damage, and also revealed omissions of the left construction due to the induction of an egocentric USN. This suggests that, although it is difficult to determine the presence or degree of USN in patients with right-sided brain injury based on their scores in the ROCF tracing task, calculating LI might more clearly reveal the presence or absence of USN, particularly the egocentric aspect of USN, which has traditionally been determined by cancellation tests or copying tasks [39].

In the ROCF copying task, there were few overlapping in all the three groups. However, in the tracing task, more overlapping on the right side was observed in the USN– group than in the healthy group, and in the USN + group than in the USN– group. Wojciulik et al. [17, 18] conducted a cancellation test in patients with right–brain damage under conditions that did not leave a trace, and they found a stronger degree of USN with that test than with the conventional cancellation task with a trace, as well as the repeated canceling of items on the right side that had already been canceled; they suggested that this was due to a spatial working memory deficit. In addition, Malhotra et al. [40] reported that poor performance in spatial working memory tasks correlated with the severity of USN in cancellation tasks. Moreover, Wansard et al. [41] reported that a group of patients with USN in a computerized task using a touch screen exhibited impaired spatial working memory and many re-cancellations. In addition to deficits in spatial working memory, the involvement of spatial remapping deficits has also been reported as a cause of such phenomena occurring in USN [42]. Many previous studies examined cancellation tasks without leaving tracing lines, and in our ROCF tracing task, we used a single figure that did not require exploration, so it seems unlikely that spatial remapping deficits would be affected, and overlapping of the left and right structures is thought to be caused by a deficit in spatial working memory. In addition, the USN + group showed a significantly greater overlapping score for the left side than in the control group, and the left score in the USN − group was slightly greater than that in the controls. Rode et al. proposed the term “hyperschematia” for the excessive writing and leftward expansion in left space in drawing and copying in right brain-damaged patients, and considered that a leftward relaxation of the spatial medium might be involved and might not be related to the USN [43]. Therefore, in addition to deficits in the spatial working memory, certain aspects of “hyperschematia” might have influenced the present results of greater overlapping scores on the left side of patients with right-brain damage.

Conventional USN screening tests and tracing tasks in patients with right-brain damage showed no clinically significant correlations. This suggests that the ROCF tracing task may reveal USN symptoms that cannot be revealed in the copying task. The fact that overlapping scores in ROCF tracing task did not show clinically meaningful correlations with the other tests may be because the overlapping scores might focus on the spatial working memory, which was not tested in the conventional USN screening test.

ROC curve analysis of the ROCF tracing task and conventional USN screening test showed moderately accurate AUC only for the ROCF tracing LI and the highest sensitivity for the tracing total overlapping score. Conventional USN screening tests have been noted to have low sensitivity and high specificity [44], and this was also true for the results of conventional screening tests in this study. The ROCF tracing LI and overlapping score, which recognized high sensitivity and AUC, were expected to be effective in reducing missed USN when combined with conventional screening tests.

These results suggest that ROCF tracing LI produced a load on spatial working memory and had power to detect egocentric USN that was not evident in the line cancellation test, the star cancellation test, or the copying task, and that the total overlapping score could detect USN with high sensitivity by assessing spatial working memory impairment. It was also suggested that the left overlapping score may reveal hyperschematia in patients with USN, which was previously thought to be non-comorbid [43].

In addition, this study used tasks with paper and pencil (and chopsticks) that were used in everyday clinical situations, which was considered a strength as it can be easily used to detect USN with greater sensitivity than conventional tests, and assess spatial working memory in USN in any clinical situation simply and conveniently.

A limitation of this study is that, as it was a cross-sectional study, the timing of onset in patients with right-side brain damage varied from subacute to chronic and it is not possible to determine how findings in the tracing task would change with improvements in USN. In the future, the mechanistic aspects of USN in the ROCF tracing task should be clarified in more detail by examining correlations between changes over time in the ROCF tracing task as well as other USN assessment tests and ADL. In addition, this study did not examine associations between brain lesions and scores in the ROCF tracing task. Future studies analyzing these associations might allow verification of what the ROCF tracing task can reveal about the mechanism of USN. Another limitation of the study is that, due to the small sample size, cutoff values for each score of the ROCF tracing task could not be established with precision.

In summary, the ROCF-based tracing task has the potential to reveal the presence of USN and the impairment of spatial working memory for existing copying tasks. In conclusion, the ROCF tracing task could be a convenient and highly detectable novel method for assessing USN.

## Data Availability

Anonymized data not published within this article will be made available by request from any qualified investigator.
